# Original Locking Rod System Designed for Diaphyseal Fractures of Long Bones

**DOI:** 10.3390/jfb16090348

**Published:** 2025-09-15

**Authors:** Liviu-Coriolan Misca, Cristian Constantin Croicu, Adrian Emil Lazarescu, Mihai-Alexandru Sandesc, Jenel Marian Patrascu, Sorin Florescu, Jenel Marian Patrascu

**Affiliations:** 1 Department of Orthopedics and Traumatology, Victor Babes University of Medicine and Pharmacy, 300041 Timisoara, Romania; liviu.misca@umft.ro (L.-C.M.); msandesc19@gmail.com (M.-A.S.); jenel.patrascu@umft.ro (J.M.P.J.); florescu.sorin@umft.ro (S.F.); patrascu.jenel@umft.ro (J.M.P.S.); 2 Department Orthopaedics and Traumatology, Cork University Hospital, T12 DC4A Cork, Ireland; 3 Veterinaria Timisoara, 300502 Timisoara, Romania; cristiancroicu@yahoo.com; 4 Spitalul Clinic Judetean de Urgenta “Pius Brinzeu” Timisoara, 300723 Timisoara, Romania

**Keywords:** intramedullary fixation, locking rod, biomechanical testing, shaft fracture

## Abstract

**Introduction**: Intramedullary nailing is widely used for long bone fractures. Traditional systems are reliable, but they present some complications regarding lack of modularity or possible growth plate damage. **Methods**: A novel locking rod–screw system featuring a central rod and a grooved screw with a secondary interlocking mechanism was developed and tested. Mechanical testing followed ASTM F543 and ISO 6475 standards using a 3.0 mm steel alloy prototype. **Results**: The system withstood mechanical testing >200,000 cycles at loads up to 200 N with no rates of failure or loosening, significantly outperforming other implants of the same size (3.0 mm TENS). **Conclusions**: The proposed implant demonstrates superior biomechanical performance in vitro, enabled by its unique hollow screw and secondary locking configuration. This modular and minimally invasive system shows promise for use in cases of smaller long bones, personalized paediatric fractures, and all types of diaphyseal fractures, but does warrant in vivo validation.

## 1. Introduction

Diaphyseal fractures of long bones, such as the femur, tibia, and humerus, pose significant challenges in orthopaedic trauma due to their role in mobility and weight bearing [1,2]. Over recent decades, intramedullary (IM) fixation has emerged as the preferred treatment due to its biomechanical advantages, including the preservation of the fracture haematoma and supporting minimally invasive fixation techniques. These are features shared by most modern intramedullary systems [3].

Although IM nailing has largely replaced plating-technique fixation in diaphyseal orthopaedic trauma clinical practice, it is not without limitations. Current IM systems often rely on fluoroscopy-guided distal locking, which increases operative time and radiation exposure, and may lead to implant failure or difficulties in osteoporotic bone [4]. Failure of distal locking screws has been reported in up to 6–10% of cases, while complications related to canal mismatch and alignment remain concerns, particularly in paediatric or osteoporotic patients where revision rates can reach as high as 15% [5,6,7].

Recent innovations have sought to reduce these complications through hybrid or self-locking systems [8,9,10]. However, such systems often present anatomical constraints, cost barriers, or high technical demands. The need persists for more modular, mechanically stable, and user-friendly alternatives.

In response, we introduce a novel IM implant featuring a central rod and a hollow locking screw equipped with a secondary internal locking mechanism. This construction resists axial and rotational displacement without relying on endosteal contact. The system is intended to offer flexibility for both human and veterinary use, and its design allows for minimally invasive assembly across varied anatomical contexts.

The current study biomechanically evaluates the proposed locking rod system under conditions replicating clinical loading scenarios. Tests simulate axial, torsional, and combined cyclic stresses to assess its mechanical viability [11,12,13,14]. The aim is not to claim clinical superiority, but rather to provide foundational data supporting future in vivo validation [15,16,17].

We give particular attention to settings where traditional nails may be less effective: narrow intramedullary canals, paediatric and osteoporotic bone, and resource-limited environments. The current study focuses on mechanical feasibility and fatigue resistance under cyclic loading, proposing the system as a promising alternative in specific fracture contexts.

## 2. Materials and Methods

### 2.1. Hardware

**Figure 1:**
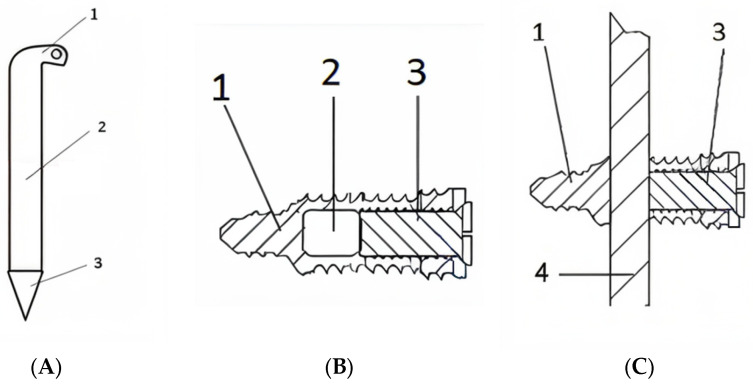
Components of the locking rod osteosynthesis system. (**A**)**:** **Central rod. 1:** The rod is bent at the proximal end to avoid plunging inside the intramedullary canal and designed with an orifice for extraction, if needed. **2:** Body of central rod with variable diameters. **3:** Distal sharp tip to facilitate passing the rod through the locking systems. (**B**)**: Locking screw model. 1:** The tip of the screw that anchors in the distal cortex from insertion point. **2:** Transverse hole, designed for the passing of the central rod through the locking system (this transverse passing hole needs to be centred with the centre of the centromedullary canal). **3:** Central secondary screw, which is advanced through the hollow main screw to lock the central rod. (**C**)**: Assembled locking rod osteosynthesis system: 1:** The tip of the locking screw (with a smaller diameter for distal cortex fixation). **3:** Central secondary crew used for locking the construction. **4:** The central rod.

**Figure 2:**
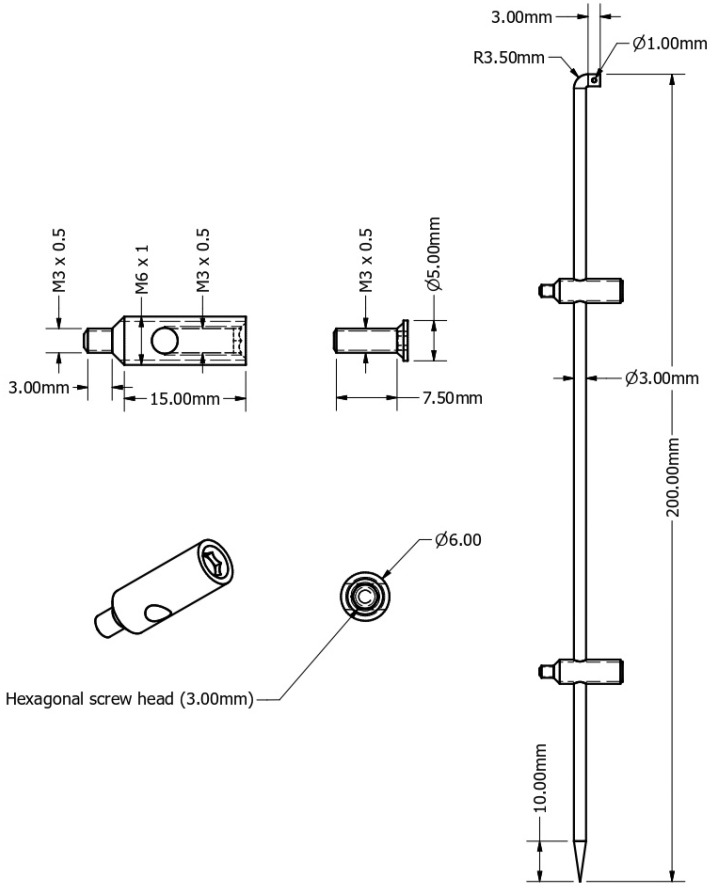
Technical drawing of the outer screw, of the inner screw and of the rod assembly.

The locking rod system contains two major components: a central titanium/steel alloy rod and multiple locking screws (specifically designed with a hollow core and a secondary internal rod locking screw that, when tightened as a final step in the surgical technique, locks the rod) (Figure 1 and Figure 2).

The central rod is available in multiple diameters and lengths in order to fit the intramedullary canal of the long bone that is fixed, and also to address different diaphyseal fractures of long bones in an optimal assembly manner in accordance with preoperative and intraoperative measurements.

At the proximal end, the rod is bent and designed with an orifice in order to facilitate extraction when and if required. At the level of the rod’s distal end, the tip of the rod is sharp; the reasoning for this specific technical tweak is to facilitate the passing of the rod through the locking screw’s transverse hole (Figure 1A).

The locking screws are designed with a hollow centre; this allows the passing of the central rod through a transverse hole from the middle region of the screw. Then, a central secondary screw is tightened inside the hollow core of the screw in order to mechanically lock the central rod as the final step of the assembly of the locking rod system (Figure 1B).

The locking screws are available in different dimensions in order to fit the optimal selected rod. Another key point for the availability of different lengths for screws and different diameters for the central rods is for proper fixation regarding the diameter of the canal and the thickness of the bone cortices that are addressed for fixation using this innovative design.

### 2.2. Surgical Technique

Preoperative planning is a key factor that needs to take the following into consideration:

1. The diameter of the bone canal to determine the optimal diameter of the central rod.
2. The length of the bone and fracture site for the optimal selection of the central rod.
3. The fracture pattern and the anatomical region to determine the final osteosynthesis.

The final locking rod assembly can be fixed using two locking screws (one screw fixed proximal and one screw fixed distal to the fracture site).

The assembly can use four locking screws (two screws placed proximal and two screws fixed distal to the fracture site). This can be useful in situations where more stability is required, either due to the fracture pattern or the forces of muscle pull given the anatomical forces that require counteraction in the postoperative period.

The diameter of the central rod determines the diameter of the locking screws, while the length of the locking screws depends on bone thickness, according to preoperative measurements.

The surgical approaches utilised for rod insertion are minimally invasive and are essentially the classical approaches for the intramedullary nailing of shaft fractures.

The surgical approach used for the placement of the locking systems depends mostly on fracture location and pattern. However general guidelines have already been established in regard to safe zones for external fixator pin placement, which can be safely used for the placement of locking screws in order to avoid neurovascular complications.

The careful dissection and good visualisation of the cortex is essential in order to correctly centre the locking screw in such a manner that the transverse hole used to pass and lock the central rod is correctly placed in the centre of the bone canal.

Proper preoperative planning will establish the optimal dimensions of the central rod and of the locking screw system, and will also determine the number of locking screws required in order to assure optimal placement for maximal stability of the construction.

### 2.3. Surgical Procedure Steps

Step 1

The first step commences with fixation of the locking screws in the proximal and distal bone fragments in accordance with the specific clinical situation. Screw fixation is carried out under image intensification control.

Precise screw placement is essential in order to place the screw’s transverse hole to the centre of the intramedullary canal. However, deviations of 1 mm to 2 mm in alignment are insignificant and will not compromise the next surgical step given the fact that the sharp distal end will allow the rod to slide into and pass through the transverse hole of the locking screws.

Step 2

Passing the central rod through the transverse hole of the locking screw can be achieved either anterograde or retrograde depending on the fracture’s location and pattern.

Reduction should be obtained through specific orthopaedic manoeuvres before attempting to pass the central rod. Image intensification is used to confirm that the rod passes through the intended locking hole.

Preoperative planning plays an essential role at this point. If the correct dimensions of the screw have been measured and coincide with the screws utilised intraoperatively, the chances that the central rod is malpositioned (between the cortex and the screw) are non-existent. The length of the screw chosen should coincide with the diameter of the intramedullary canal, and this will make it impossible to pass the rod through anything else besides the intended locking hole in the plane of screw fixation. In case of misalignment of the rod with the screw in other planes, when addressing a wider intramedullary canal, a centraliser to facilitate the passing of the rod can be used.

The correct position of the rod within the locking holes is confirmed under fluoroscopy before advancing to the next surgical step.

Step 3

**Figure 3:**
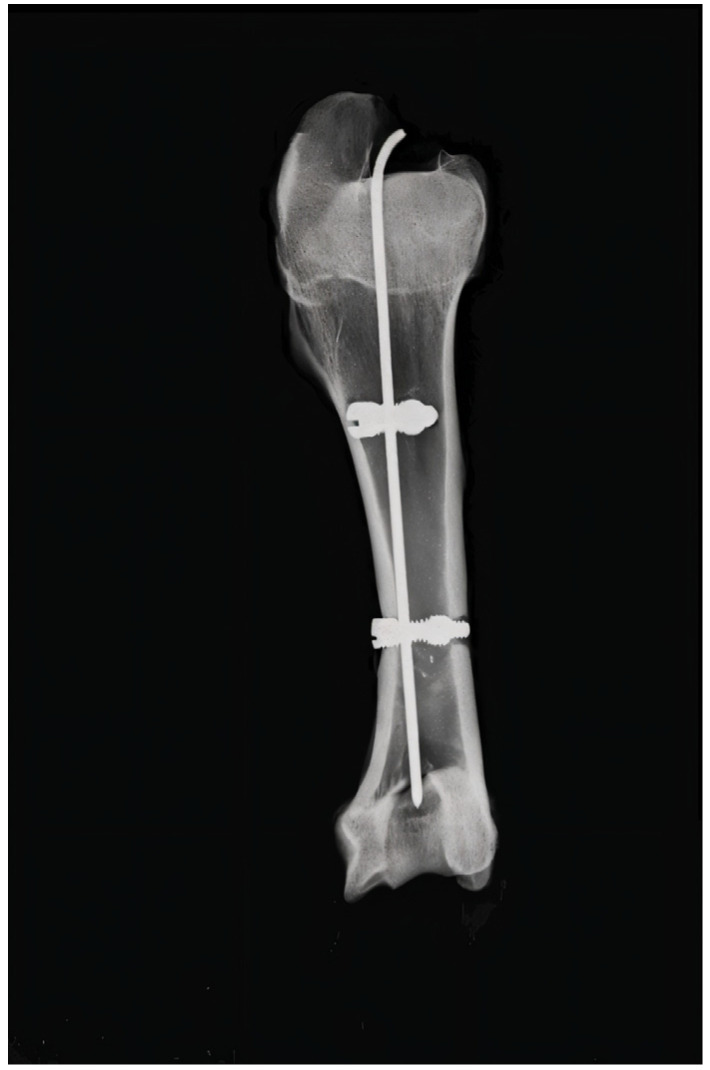
X-ray of the assembled osteosynthesis locking rod system.

Fluoroscopic confirmation of fracture reduction and rod length is pursued before locking the rod into the locking holes. If the image intensification is satisfactory, the central screw is tightened as designed in order to lock the rod and stabilise the entire osteosynthesis construction (Figure 3).

### 2.4. Prototype and Mechanical Stability

The study was conducted using a steel alloy prototype composed of a rod with a diameter of 3 mm locked into a 6 mm locking screw system. The rod was manufactured in the laboratories of the Politehnica University of Timisoara, while the locking system was hand-made by the inventor of the system.

The screws used in the current study were manually manufactured for the purposes of prototyping. They were fabricated by hand from medical grade 316 L stainless steel rods, using precision manual tools for cutting, threading, and shaping. This approach allowed for rapid iteration and customization during the early developmental phase of the locking rod system. The handmade screws were produced with careful attention to dimensional tolerances to ensure mechanical function and construction stability.

Stainless steel (316 L) was selected for the prototype due to its ease of manual fabrication, availability, and cost effectiveness, allowing rapid prototyping. This facilitated early testing before transitioning to titanium, which is better suited for clinical-scale production.

The prototype locking rod system was tested mechanically in the CIDUCOS Laboratory of Politehnica University of Timisoara in Romania. The mechanical testing of the prototype system was performed using a pulsatory testing machine (INSTRON 8800 manufactured by Ro-Mega Control SRL, Bucharest, Romania) on the locking rod system fixed in a fractured bone (of animal origin). The fractured bone was fresh-frozen porcine humerus (obtained from a licenced local abattoir in Timisoara, Romania; the animals were processed for the food industry, and no animals were sacrificed specifically for this study), which was thawed at room temperature for 12 h prior to testing. All surrounding soft tissues had been removed. The testing protocol was intended to replicate the real scenario for this innovative system in veterinary and human orthopaedic surgery.

The Instron device is considered a gold standard device for biomechanical testing due to its high precision, repeatability, and control over the applied forces. The Instron device facilitates the accurate measurement of force, displacement, and mechanical properties, ensuring consistency in data collection.

The testing setup on the Instron device replicates physiological forces experienced by intramedullary nails or rods during fracture healing and weight-bearing activities; by applying controlled loading forces, biomechanical studies performed with the device can mimic the mechanical stress encountered in clinical applications [18].

Mechanical load testing of the locking rod construction was conducted in multiple loading conditions, with each condition selected for its biomechanical relevance:

1. Tensile–compression test: This type of testing simulates axial loading, which is experienced in a clinical setting during weight-bearing activities, walking, or running. Tensile–compression testing determines the implant stiffness, elastic modulus, and load-bearing capacity of an implant. A well-fixed fracture site using an intramedullary rod should exhibit minimal axial displacement and high stiffness; the excessive deformation of the implant or fracture fragments would suggest implant instability or material fatigue [19].
2. Torsion testing: This test evaluates the rotational stability of the implant and the efficiency of the locking mechanism. Torsion testing simulates the twisting forces encountered during gait, turning, accidental falls or rotational movements. High torsional stiffness indicates good rotational fixation, while excessive rotational displacement might suggest loosening of the locking mechanism or implant failure [20].

Torsional load was applied using the built fixture that aligned the rod’s longitudinal axis with the torque axis. One end of the construction was fixed, while the opposing end was rotated using the calibrated motor. Torque was measured in real time using a load cell aligned coaxially with the rod–bone construction.

3. Combined torsion–tensile–compression testing: This specific test mimics realistic in vivo conditions, where multiple forces act simultaneously, this type of testing helps assess the complex stress interactions in fracture fixation systems. Combined torsion–tensile–compression testing can identify the stress concentration points where implant failure is most likely to occur and also provides insight into implant fatigue under complex load cycles [21].
4. Three-point bending testing: This type of test was conducted in order to evaluate flexural strength and fatigue resistance. The three-point bending test simulates direct impact forces from falls or external trauma. High bending stiffness without secondary displacement of the fracture indicates a mechanically stable implant, while low resistance during bending testing suggests implant fragility or material deficiencies [22].

A stainless steel alloy rod (316 L stainless steel—a non-magnetic, low-carbon austenitic alloy used commonly in orthopaedic surgery; its composition includes approximately 16–18% chromium, 10–14% nickel, 2–3% molybdenum, and <0.03% carbon, offering high corrosion resistance and biocompatibility) was used for biomechanical testing due to its high fatigue resistance and yield strength. Stainless steel alloy implants provide better initial fixation stability compared to titanium [23].

The system manufacturing characteristics of the original locking rod system followed the guidelines of the international standard set by ISO 5832: 2016 [24].

Mechanical tests were designed in accordance with the general principles of ISO 6475:1989 [25] and ASTM F543:2017 [26] using axial loads between 150 and 250 N and torsional loads of 0.5–1.0 Nm to simulate forces encountered during the stance and midstance phases of the gait cycle. Although a fully standardised fatigue protocol was not applied, these loads were chosen based on prior biomechanical studies to reflect clinically relevant cyclic conditions in long bones [27].

**Figure 4:**
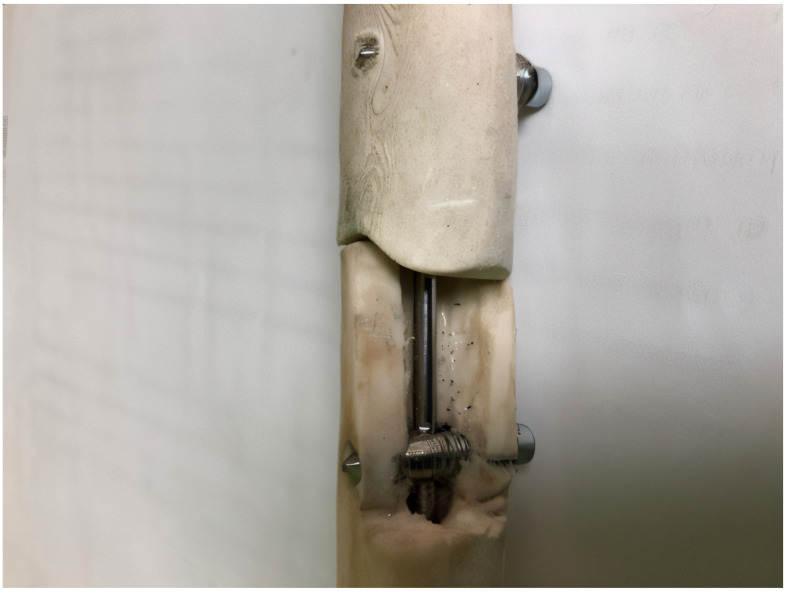
Steel alloy locking rod system fixed in fractured bone (bone window present).

Photos of the experimental model used during mechanical testing (for axial and rotational stability in a long bone shaft fracture fixed by this construction) were taken in order to present the correct placement of the assembly. A bone window was created to exemplify the positioning of the central rod in the intramedullary canal (Figure 4).

**Figure 5:**
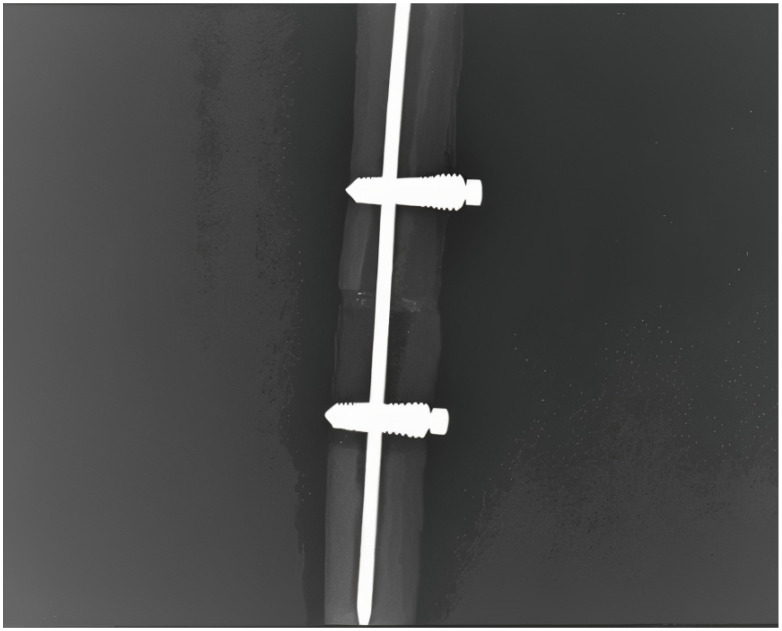
X-ray of steel alloy locking rod system fixed in fractured bone (bone window present).

An X-ray of the same experimental model was performed in order to demonstrate the correct locking screw position and the optimal assembly of the system (Figure 5).

Mechanical testing was performed in vitro by applying different types of forces to the prototype construction: tensile compression; torsion; tensile compression and torsion; and three-point bend. The testing frequency measured between 0.5 Hz for three-point bend forces and 10 Hz for tensile compression forces.

The assembled prototype was exposed to mechanical loads between 70 N and 200 N during testing, and the torsion forces measured 0.8 Nm in the case of combined forces and 1.0 Nm when torsion testing was performed alone.

## 3. Results

Mechanical testing of the assembled locking rod system was performed for a number of cycles in order to replicate the mechanical stress during a hypothetical rehabilitation period until bone healing is expected.

The tensile–compression force mechanical testing was conducted with two settings of bone contact: a 1 mm gap between the fragments and with fragments in physical contact. Torsion, combined tensile–compression and torsion, and three-point bend stress testing was only performed with fragments in physical contact.

Stress testing on the osteosynthesis construction was performed for a set number of cycles and not until failure of the implant, and all of the preset number of stress testing cycles were achieved without failure. All experimental cycles were performed on the same implant construction.

The mechanical fatigue stress testing results are described for all four types of mechanical testing below.

1. Tensile compression testing was performed in two scenarios:
  - Bone in physical contact with a mechanical load of 200 N (with a preloading force of 50 N). The number of cycles in this setting was 200,000.
  - Bone with a 1 mm gap at the level of the fracture site with a mechanical load of 200 N (with a preloading force of 50 N). The number of cycles in this setting was 100,000.
2. Torsion testing was performed with the fracture fragments in physical contact with a torque force of 1.0 Nm. Torsion testing was repeated for 50,000 cycles.
3. Combined tensile–compression–torsion testing was performed with the fracture fragments in physical contact with a torque force of 0.8 Nm and a tensile force of 200 N. The number of cycles for this test was 150,000.
4. Three-point bend testing was performed with the fracture fragments in physical contact with a mechanical load of 70 N. The number of cycles for this test was 30,000.

The results of the testing are presented in the following table (Table 1).

Under cyclic axial loading of 200 N, the construction showed <0.01 mm displacement, corresponding to an axial stiffness of ≈20,000 N/mm. Torsional testing (1.0 Nm) resulted in <0.1° rotation, corresponding to ≈573 Nm/° stiffness. In the three-point bending test (70 N), the deflection was <0.02 mm, yielding ≈ 3500 N/mm flexural stiffness. Combined axial-torsional loading likewise resulted in displacements below the device’s sensitivity threshold.

The data is highly suggestive that this osteosynthesis device supports good axial and rotational stability, properties that are not lost in time due to repetitive forces applied on the fixed bone.

## 4. Discussion

This study evaluated a novel 3.0 mm stainless steel locking rod system for long bone diaphyseal fractures, showing strong performance in cyclic mechanical testing. The system endured over 500,000 loading cycles without implant failure or loosening, suggesting notable fatigue resistance for its size.

Compared to conventional devices, the system exhibits distinct mechanical advantages. In a prior publication by Mahar et al., 3.0 mm titanium elastic nails showed limited torsional and axial stability, instability at the fracture site, and low compressive strength [28]. In contrast, the studied system withstood 150,000 combined load cycles (0.8 Nm torsion, 200 N compression) and 200,000 axial load cycles with bone in contact. Even with a 1 mm fracture gap, 100,000 cycles of axial loading were completed at 200 N. This indicates improved load transfer and construction integrity under stress.

The three-point bending results also demonstrated high fatigue resistance. While headless intramedullary screws failed at compression around 215 N in similar diameter applications, the studied system withstood 30,000 bend cycles at 70 N without damage, even though no failure load was measured [29,30].

To extrapolate clinical utility, we applied material and scaling theory. Maintaining equivalent bending stiffness when switching from steel to titanium would require a ≈7% increase in rod diameter [31,32,33,34]. Our calculations suggest that steel rods ≥ 5.3 mm or titanium rods ≥ 6.2 mm could match the strength needed for adult femoral IM fixation. This scalability aligns with clinical data on rod sizing for large bones [Appendix A].

Material properties further support the system’s adaptability. Titanium offers superior fatigue resistance and reduces stress shielding due to lower stiffness, while stainless steel offers higher initial rigidity [33]. Each material has specific clinical advantages, and the system’s modular design allows for patient specific selection.

A key biomechanical distinction is the absence of endosteal contact. Traditional IM nails rely on canal press-fit, which can limit flexibility and increase the risk of canal mismatch or over-reaming. The studied rod’s design locks the rod via a transverse screw passage and internal screw fixation, enabling fixation in wide or narrow canals while maintaining the construction’s rigidity [35,36,37]. This may reduce stress shielding and promote micro-motion.

Dynamic fixation is also achievable. Stiffness can be modulated by adjusting the number and position of the locking screws or by selectively loosening one end of the construction; this provides intraoperative flexibility.

The system’s design is well suited to simple, segmental, or comminuted fractures. Its load-bearing capacity is independent of bone contact, relying instead on the screw–rod construction. A four-screw configuration (two proximal, two distal) creates a bridging mechanism that maintains alignment across bone loss zones [38]. The modularity of the rod and screw lengths enables adaptation across various bones—from metacarpals to femurs—based on anatomical shaft diameters [39,40].

From a surgical perspective, the design simplifies distal locking. Traditional freehand fluoroscopy-guided locking is both technically demanding and radiation-intensive. Here, the screw–rod interface is pre-aligned and mechanically integrated, potentially reducing operating time and fluoroscopy use. Rods are cut to length intraoperatively and, by eliminating the need to stock multiple sizes, offer economic and logistical advantages. While this system avoids canal reaming and allows for controlled rod insertion, the need for cortical exposure to insert the locking screws may slightly increase dissection requirements compared to conventional nail systems.

Challenges include the accurate alignment of the rod through screw holes during insertion. This may be overcome by centralising tools or reduction instruments, similar to those used in minimally invasive procedures [41]. These holes support easier passage of the rod through pre-fixed locking screws and ensure construction centrality.

Our testing approach also replicated clinically relevant fracture conditions, including 1 mm fracture gaps and combined cyclic loading stress testing. The system endured all of the mechanical tests without displacement or plastic deformation, indicating mechanical reliability during the early phases of stability required in fracture fixation when the implant bears full stress.

Despite the strengths of this study, limitations remain. Only one prototype, 3.0 mm in diameter, was tested and the study was performed in vitro using animal bone models. Biological integration, healing timelines, and osteogenesis were not assessed during this study.

Only one rod diameter (3.0 mm) was tested, and the screws were manufactured using handmade techniques. This introduces variability in precision and tolerances compared to industrial manufacturing, representing a limitation in reproducibility. Future work will therefore incorporate multiple rod sizes and industrially fabricated components to ensure consistent mechanical performance.

A limitation of the present study is that a statistical analysis of the results (mean ± standard deviation or comparative testing) was not possible, as only individual handmade prototypes were available for mechanical evaluation. Future studies using standardised, industrially manufactured constructions and larger sample sizes will allow rigorous statistical analysis to be performed.

Furthermore, although a comparison to literature data suggests strong mechanical performance, direct head-to-head testing with commercial IM nails has not yet been performed.

Future directions include scaling up the system for adult femoral and tibial applications, refining the alloy composition and conducting in vivo validation. Anatomical variations such as femoral bowing or the specificity of entry into the tibial centromedullary canal may present challenges to rod insertion in specific cases. These variations might require further design optimisation in future iterations of the system. Clinical trials would be required to confirm biological performance and safety in human patients.

In addition to biomechanical validation, long-term implant performance will depend on biological responses, including bone healing dynamics and osteointegration. These aspects can only be addressed through in vivo models, which will represent the next stage of evaluation. Translational development will also require compliance with international regulatory standards (ISO 5832 for metallic biomaterials and ASTM F1264:2016 [42] for intramedullary fixation devices), preclinical animal testing, and, ultimately, controlled clinical trials.

While the construction’s mechanical performance is encouraging, the absence of biological validation and limited prototype testing confirm that the system is still in its early conceptual stage. These findings must be interpreted as a preclinical feasibility analysis, and should not be overextended towards clinical efficacy.

## 5. Conclusions

This study presents a novel locking rod system, which can be used for diaphyseal fracture fixation, that demonstrates excellent axial and torsional stability under repetitive mechanical loading. The prototype withstood high-cycle tensile, torsional, and combined mechanical stresses without failure, indicating strong fatigue resistance even at a central rod diameter of 3.0 mm. Unlike conventional intramedullary nails that require endosteal contact, this system achieves fixation through the locking screws, allowing for minimally invasive application and potential biological advantages such as reduced stress shielding and the preservation of fracture haematoma (features shared by most modern intramedullary systems). Its modular design—with different options for varying rod diameters and screw lengths—makes this system adaptable across a range of long bones, including for paediatric, adult, and veterinary use.

However, these promising results are based solely on in vitro biomechanical testing. No in vivo evaluation of osteogenesis, healing timelines, or biological integration has yet been performed. Future studies must address these gaps through scaled-up biomechanical models, animal trials, and clinical studies. If biological performance aligns with mechanical findings, this system could offer a cost-effective, flexible, and less invasive alternative to standard intramedullary nails, particularly in cases requiring immediate mobilisation or customised fixation strategies. Its ability to provide both static and dynamic stability through simple intraoperative adjustments positions it as a potentially valuable tool in modern orthopaedic trauma care.

## 6. Patents

The locking rod system is patented by Cristian Constantin Croicu at the Romanian State Office for Inventions and Branding, Patent Number 132187/30.07.2021 (patent valid for 20 years from 20 June 2017).

## Figures and Tables

**Table 1:** Stress mechanical testing of the locking rod system.

Type of Mechanical Testing	Bone Contact	Frequency [Hz]	Mechanical Load	Imposed Number of Cycles (Not to Failure)	Displacement	Stiffness
Tensile–compression	In physical contact	10	200 N (−50 N preloading)	200,000	<0.01 mm	≈20,000 N/mm
	1 mm gap between bones	5	200 N (−50 N preloading)	100,000	<0.01 mm	≈20,000 N/mm
Torsion test	In physical contact	5	1.0 Nm	50,000	<0.1° rotation	≈573 Nm/°
Combined testing (tensile–compression–torsion)	In physical contact	5	Torsion: 0.8 Nm Tensile: 200 N	150,000	<0.01 mm and <0.1° rotation	≈20,000 N/mm and ≈573 Nm/°
Three-point bend	In physical contact	0.5	70 N	30,000	<0.02 mm deflection	Flexural stiffness ≈3500 Nm

## Data Availability

The original contributions presented in the study are included in the article, further inquiries can be directed to the corresponding author or to the main authors.

## Abbreviations

The following abbreviations are used in this manuscript:

IM	Intramedullary
Fig	Figure
mm	Millimetres
N	Newton
Nm	Newton-metre
E	Young’s modulus
G	Shear modulus
I	Moment of inertia
D	Diameter
π	≈3.14

## Appendix A

A comparison of the material properties of titanium and steel alloy used in orthopaedics is presented in the following table (Table A1).

**Table A1:** Comparison of titanium and steel alloy material properties.

Property	Titanium (Ti-6Al-4V)	Stainless Steel (316 L)
Young’s Modulus (E)	~110 GPa	~200 GPa
Shear Modulus (G)	~44 GPa	~77 GPa
Density	~4.4 g/cm^3^	~8.0 g/cm^3^
Tensile Strength	~1000 MPa	~570 MPa
Fatigue Strength	Higher	Lower
Corrosion Resistance	Excellent	Moderate
Stress Shielding Risk	Lower (better load sharing)	Higher (stiffer implant)

In order to optimize diameter (*D*) of the central rod based on material stiffness and on the load bearing requirements, we can derive the following from the fundamental bending stiffness equation (*EI* = constant) using *E* (Young’s modulus) and the moment of inertia (*I*) [31]:

$$I=\frac{\pi }{64}D^{4}$$

Since the bending stiffness should remain the same with the goal of changing the rod material from steel alloy to titanium, $E_{steel}I_{steel}=E_{titanium}I_{titanium}$

$$E_{steel}D_{steel}^{4}=E_{titanium}D_{titanium}^{4}$$

$$D_{titanium}=D_{steel}\times (\frac{E_{steel}}{E_{titanium}}{)}^{1/4}$$

$$\frac{E_{steel}}{E_{titanium}}=\frac{200}{110}\;\approx 1.82$$

$$D_{titanium}=D_{steel}\times (1.82{)}^{1/4}$$

$$D_{titanium}\approx D_{steel}\times 1.17$$

The equation proves that the diameter for a titanium rod should be at the least approximately 17% larger than a steel rod in order to maintain the same bending stiffness.

In order to preserve the same resistance of material tested in this current study on a 3.0 mm steel alloy diameter rod, it would be necessary to use a titanium rod of at least 3.5 mm in diameter.

The bending stiffness of an intramedullary rod depends on the Young’s modulus of the material and the second moment of inertia of the rod’s cross section.

For a cylindrical solid central rod,

$$I=\frac{\pi }{64}D^{4}$$

Since bending stiffness is proportional to *D^4^*, the required diameter for a central rod used in a femoral shaft fracture can be estimated by

$$D_{femur}=D_{3mm}\times {\left(\frac{M_{femur}}{M_{3mm}}\right)}^{\frac{1}{4}}$$

where

- $D_{3mm}$ = 3 mm (rod diameter tested biomechanically);
- $M_{femur\;}$ = average bending moment in femoral shaft fractures (which is described in the literature to be approximately 10 to 12 times greater in fractures of the femoral shaft compared to metacarpal fractures);
- $M_{3mm}$ = bending moment of the tested 3 mm central rod.

Using the conservative estimate of 10× the load requirement, the calculation would be

$$D_{femur}=3\;mm\times {\left(10\right)}^{\frac{1}{4}}$$

Approximating $10^{\frac{1}{4}}\approx 1.78$

$$D_{femur}\approx 3\;mm\times 1.78=5.3\;mm$$

Thus, a central rod used for femoral shaft fixation should be at least 5.3 mm in diameter in order to provide comparable biomechanical resistance. These calculations are based on the results acquired from testing the central rod of 3 mm in diameter.

Applying the calculations for other materials, such as titanium rods,

$$D_{titanium}\approx D_{steel}\times 1.17$$

The equivalent titanium central rod diameter would be at a minimum of

$$D_{titanium}\approx 5.3\;mm\times 1.17=6.2\;mm$$

Thus, the titanium central rod should have a diameter of at least 6.2 mm in order to provide sufficient fixation for femoral shaft fractures.

This theoretical calculation can be applied to various diameters taking into account different diameters of centromedullary canals that are found in different long bones.

For a 6 mm central rod, the internal transverse passage within the locking screw must have a diameter of approximately 6.2 mm, allowing for smooth passage with slight clearance.

The outer diameter of the locking screw would need to be at least 8.9 mm. This would ensure that the screw walls retain sufficient thickness (≈1.35 mm per side) to maintain strength and resist torsional forces while not over-sizing the implanted screw.
